# Host stress hormone norepinephrine stimulates pneumococcal growth, biofilm formation and virulence gene expression

**DOI:** 10.1186/1471-2180-14-180

**Published:** 2014-07-04

**Authors:** Sara Sandrini, Fayez Alghofaili, Primrose Freestone, Hasan Yesilkaya

**Affiliations:** 1 Department of Infection, Immunity and Inflammation, University of Leicester, University Road, Leicester LE1 9HN, UK

## Abstract

**Background:**

Host signals are being shown to have a major impact on the bacterial phenotype. One of them is the endogenously produced catecholamine stress hormones, which are also used therapeutically as inotropes. Recent work form our laboratories have found that stress hormones can markedly increase bacterial growth and virulence. This report reveals that *Streptococcus pneumoniae*, a commensal that can also be a major cause of community acquired and nosocomial pneumonia, is highly inotrope responsive. Therapeutic levels of the stress hormone norepinephrine increased pneumococcal growth via a mechanism involving provision of iron from serum-transferrin and inotrope uptake, as well as enhancing expression of key genes in central metabolism and virulence. Collectively, our data suggests that *Streptococcus pneumoniae* recognises host stress as an environmental cue to initiate growth and pathogenic processes.

**Results:**

Effects of a clinically attainable concentration of norepinephrine on *S. pneumoniae* pathogenicity were explored using *in vitro* growth and virulence assays, and RT-PCR gene expression profiling of genes involved in metabolism and virulence.

We found that norepinephrine was a potent stimulator of growth, via a mechanism involving norepinephrine-delivery of transferrin-iron and internalisation of the inotrope. Stress hormone exposure also markedly increased biofilm formation. Importantly, gene profiling showed that norepinephrine significantly enhanced expression of genes involved in central metabolism and host colonisation. Analysis of the response of the pneumococcal *pspA* and *pspC* mutants to the stress hormone showed them to have a central involvement in the catecholamine response mechanism.

**Conclusions:**

Collectively, our evidence suggests that the pneumococcus has mechanisms to recognise and process host stress hormones to augment its virulence properties. The ability to respond to host stress signals may be important for the pneumococcal transition from colonization to invasion mode, which is key to its capacity to cause life-threatening pneumonia, septicaemia and meningitis.

## Background

*Streptococcus pneumoniae* is a major cause of otitis media, meningitis, septicaemia and community and hospital acquired pneumonia [1]. As well as being a potentially deadly pathogen, the pneumococcus often resides in the human nasopharynx without causing harm, a situation known as carriage. Therefore two fundamental but so far unanswered questions arise from consideration of pneumococcal carriage and the range of diseases it causes. Which host signals trigger transition of the pneumococcus to a pathogenic state, and how does the bacterium sense, process and respond to these signals during the infection in order to modulate its virulence in different tissue sites?

Increasing numbers of studies of infectious bacteria are suggesting that the neuroendocrine (stress hormone) status of a host may determine the outcome of an infection [2]. The recognition that stress hormone release leads to increased risk of infection has come from the finding that stress –associated chemicals negatively modulate immune function [3], and to their impact on the growth and virulence of bacteria [2] Catecholamines have been shown to augment the growth of species including *Escherichia coli*, *Salmonella typhi, Pseudomonas aeruginosa, Campylobacter jejuni* and *Bordetella bronchiseptica*[4]. The growth stimulating effect of catecholamines has been largely related to the catechol-containing moiety forming a complex with the iron within transferrin (Tf) or lactoferrin (Lf), which weakens Fe binding and so enables bacteria to acquire the normally inaccessible complexed-Fe [5]. Catecholamines have also been shown to directly modulate bacterial virulence. For instance, norepinephrine (NE) stimulated the inflammatory and secretory responses caused by *E. coli* O157:H7, and augmented the microbe’s attachment to intestinal mucosa [6]. NE increased both the cellular cytotoxicity and enterotoxicity of infection caused by *Vibrio parahaemolyticus* and up-regulated the expression of type III secretion system-1 genes [7]. Catecholamine inotropes used in the treatment of acutely ill patients (such as dopamine and epinephrine) also have been shown to increase staphylococcal and pseudomonad biofilm formation and promote recovery from antibiotic damage [2,8,9].

Most of our knowledge on bacteria-catecholamine interactions originated from the studies of Gram negative gut pathogens, and comparatively little is known about the interaction of Gram-positive bacteria with catecholamines [2,4]. In particular, the infection significance of *S. pneumoniae-*catecholamine interactions is unclear in spite of the demonstration of a significant increase in plasma stress hormone level in patients with pneumococcal pneumonia compared to healthy individuals [10]. Additionally, in an experimental mouse model of pneumococcal pneumonia it was shown that mice pre-exposed to stress were more susceptible to pneumococcal infection [11]. Very recently Marks *et al.*[12] used a tissue culture biofilm model of infection combined with animal studies to show that treatment of pneumococcal biofilms with a variety of host factors such as ATP, glucose, NE and cell lysates induced bacterial dispersal, and promoted *S. pneumoniae* colonization of normally sterile host tissues. Although this study showed that host chemicals could influence the phenotype of *S. pneumoniae*, the molecular mechanisms by which these behavioural changes were induced were not determined. In this study we show that therapeutic levels of NE can directly affect the growth and virulence of *S. pneumoniae* and identify the genes involved in host signal recognition.

## Methods

### Reagents

Human serum transferrin (Tf), ferric nitrate, and the catecholamine norepinephrine were purchased from Sigma Chemical Co. (Poole, Dorset. UK); ^55^FeCl_3_ (IES, specific activity 5 mCi/mg Fe), ^3^H-NE (TRK584,l-[7,8-^3^H] norepinephrine) were obtained from Amersham Life Sciences, UK.

### Bacterial strains and growth conditions

*S. pneumoniae* type 4 strain TIGR4, and type 2 strain D39 and its isogenic mutants were used in this work. Routinely, for inoculum preparation pneumococci were grown at 37°C in microaerophilic conditions either in brain heart infusion broth (BHI), Todd-Hewitt Broth (THB) (Oxoid, Basingstoke, UK) or on Blood Agar Base (Oxoid) supplemented with 5% (v/v) horse blood. Where appropriate the growth medium was supplemented with 100 μg/ml spectinomycin. In addition, to test catecholamine responsiveness of the pneumococci we used Sicard’s defined medium supplemented with 50% (v/v) serum-SAPI (a host-like serum-supplemented minimal medium) [2], which we refer to in the text as serum-medium. To test catecholamine responsive, bacteria were grown in the presence or absence of 10 μM NE (a concentration that Thompson *et al.* had shown to occur in the human circulation following inotrope administration) [13]. Bacteria were inoculated at approximately 10^7^ CFU/ml, and precise inoculum levels determined using pour plate counting [2]. All growth assays were carried out in at least triplicate. To prepare passaged D39, mice infected intra-peritoneally with 100 μl of overnight grown bacteria in sterile PBS. When the signs of disease were observed, blood was collected by cardiac puncture after deep anaesthesia as described previously [14,15], and 10 ml BHI was inoculated with 50 μl blood. After overnight growth, bacteria were recovered by centrifugation and then the pellet was used to inoculate 10 ml BHI containing 20% (v/v) calf serum (Sigma). When the OD_500_ reached 1.6, growth was ceased and aliquots were kept in -80°C until required.

### Mutant construction

*In vitro* mariner mutagenesis was used to introduce mutation to *pspA* as described previously [15,16]. Approximately 2 kb genomic region containing the target gene was amplified with the appropriate primers (Table 1). For transposition reactions 200 ng of PCR fragment was mixed with 200–400 ng of donor mariner plasmid pR412, which contains a spectinomycin resistance cassette, and incubated in the presence of purified *Himar1* transposase, as described previously [15,16]. Gaps in transposition products were repaired with T4 DNA polymerase (New England Biolabs, Ipswick, USA) and subsequently by *E. coli* ligase (New England Biolabs). Repaired transposition products were transformed into *S. pneumoniae* D39 using synthetic competence-inducing peptide [17]. Transformants isolated from selective medium were tested for the presence of mariner mini-transposons through PCR and sequencing [18], and then the purified products were sequenced using MP127 primer. One of the transformants designated as *pspA*^-^, was selected for further study.

**Table 1:** Oligonucleotide primers used in this study

**Primer ID**	**Primer Sequence (5’-3’)**	**Target gene in D39**
**SPD0014RTF**	GAAGGCATGCTCTGCTTACA	*comX*
**SPD0014RTR**	CGCTTCTGACTTTCCTGCTT	
**SPD0063RTF**	ATCCCAATCATCGGTGGTA	*strH*
**SPD0063RTR**	CGGTTCAGGTCTTTTGGTA	
**SPD0065RTF**	GGACCTCTTTGTAACAGGAA	*bga3*
**SPD0065RTR**	CATCTGCCAATTCCTTAGGA	
**SPD0126F**	GGAATGAAGGAAGATGATGC	*pspA*
**SPD0126R**	GCCATCTACAGTTGTGTTG	
**SPDRT0144F**	GGCGAGAAAGCTTAAGCAGA	*rgg*
**SPDRT0144R**	TTGTGCCCAAACTCATCAAA	
**SPD0344RTF**	GCAGAAAAATTGAGCCGAAC	*ritR*
**SPD0344RTR**	CGAAATACGCGCTACCAGAT	
**SPD0420RTF**	TGGTGTTTACGCACGTCTTG	*pflB*
**SPD0420RTR**	CATCAACCCCGTAAAGGTCAC	
**SPD0709RTF**	TCGTGTGGCTGCCAAGCGTG	*gyrB*
**SPD0709RTR**	GGCTGATCCACCAGCTGAGTC	
**SPD0722RTF**	CGTCACCTTCACATGACACC	*spxB*
**SPD0722RTR**	CATGTTGAATGCTCCGTCAC	
**SPD0939RTF**	CAAAATTGAAAAATGGGGCTA	*rgg/mutR*
**SPD0939RTR**	GCAAGCTGAGAGACAATCTGC	
**SPD1463RTF**	ACTCATTGTAACCAGCGAAGGAGCA	*psaA*
**SPD1463RTR**	CCCAGATGTAGGCACTTGGAACACC	
**SPD1464RTF**	AGAATTGGCTGGACTGGACAA	*tpxD*
**SPD1464RTR**	CACCGCACCAACGTTTTTG	
**SPD1499RTF**	GGAGTGAGCCAATTTTTGC	*nanB*
**SPD1499RTR**	GCAGGCATAACATCAGCT	
**SPD1504RTF**	AGCAACCTCTGGCAAATGAA	*nanA*
**SPD1504RTR**	ATAGTAATCTCTTGGAATT	
**SPD1634F**	TCTCGGTGCTCGTATGACAG	*galK*
**SPD1634R**	CACCTGCAACTTCAGCGATA	
**SPD1652RTF**	CTTTGGTGCCAAATCTCGTT	*piuA*
**SPD1652RTR**	GCAAGGGTACGGTTGATGAC	
**MP 127**	CCGGGGACTTATCAGCCAACC	*pR412* specific
**MP 128**	TACTAGCGACGCCATCTATGTG	

Construction of *pspC* mutant (*pspC*^-^) was carried out as described previously [19]. To create the *pspA* and *pspC* double mutant, the mutated region in the *pspA*^*-*^ was PCR amplified with pspAF and pspAR primers, and the amplified region transformed into *pspC*^*-*^. The mutation was confirmed as described above, and one transformant, designated *pspAC*^*-*^, was selected for further study. PspA and PspC mutants constructed in the way described above have been used in a number of different studies [16,19,20].

### Quantitative RT-PCR

The extraction of RNA from catecholamine-treated and control D39, *pspA* and *pspC* cultures (grown as described in Methods) was carried out by the Trizol method using mid-log phase cultures as described previously [21,22]. Before use the RNA was treated with amplification grade DNase I (Qiagen, Crawley, UK) and subsequently purified with an RNeasy Mini Kit (Qiagen). First strand cDNA synthesis was performed on approximately 1 μg DNase-treated total RNA using 200 U of SuperScript II reverse transcriptase (Invitrogen, Paisley, UK), at 42°C for 55 min, and random hexamers [22]. The transcription level of specific genes was normalised to *gyrB* transcription, and amplified in parallel with SPD0709RTF and SP0709RTR primers. To reduce the bias in qRT-PCR we used primer pairs with similar PCR efficiencies. The results were analysed by the comparative C_T_ method, and a 2-fold difference in expression relative to control was considered to be significant [22].

### Pneumococcal transferrin binding assays

To analyse transferrin binding to the pneumococci, overnight cultures grown as described in individual experiments (approximately 10^9^ CFU/ml) were harvested by centrifugation at 10,000 *g* for 10 min, washed twice and re-suspended in 1 ml of 100 mM Tris-SAPI pH7.5 [2,8,23]. Tf was added at 1 μg/ml; the negative control consisted of addition of an equivalent volume of distilled water. Test and control cultures were incubated at 37°C for 1 hr, after which the bacteria were centrifuged at 10,000 *g* for 10 min, washed twice in PBS and re-suspended in 100 μl of 100 mM Tris–HCl (pH 6.8) containing 10% (v/v) glycerol, 2% (w/v) sodium dodecyl sulfate (SDS), 0.1% (w/v) bromophenol blue and 100 mM dithiothreitol (DTT). This suspension was heated to 100°C for 15 min to release bound Tf. The cell free extracts were then centrifuged at 10,000 *g* for 10 min, and the supernatant electrophoresed on 10% SDS-polyacrylamide gels, and electroblotted onto PVDF membranes. Blots were probed with anti-Tf polyclonal antisera and cross-recognition was determined using HRP-conjugated secondary antibodies and enhanced chemiluminescence as described previously [23].

### Pneumococcal transferrin iron uptake

To test the ability of *S. pneumoniae* to acquire iron from Tf, serum-medium containing filter-sterilized ^55^Fe-Tf (2 × 10^5^ cpm ml^-1^) was supplemented with 10 μM NE or an equivalent volume of water (control). Washed cultures were added at 1 × 10^7^ CFU/ml and incubated at 37°C in a 5% CO_2_ atmosphere for 24 hr. For analysis of catecholamine internalisation, cultures were similarly grown but supplemented with 1 × 10^5^ cpm per ml of ^3^H-norepinephrine (control), with and without 10 μM norepinephrine. Cultures were harvested by centrifugation at 10,000 *g* for 10 min, washed in PBS and assayed for cell numbers and for radiolabel incorporation, using scintillation counting as described previously [8,23].

### Biofilm formation

Stress hormone effects on biofilm formation was analysed microscopically and using the crystal violet attachment assay [24]. Bacteria were cultured statically in serum-medium in 150 μl volumes in triplicate in 96 well plates. To ensure that growth levels of control and catecholamine-treated cultures were the same, we inoculated cultures at a higher level of 10^8^ CFU/ml. After incubation, non-attached bacteria and culture supernatants were removed and the wells washed 3 times with PBS. The wells were then were dried in hot air cabinet set at 50°C. After drying, crystal violet (0.2% v/v) was added for 15 minutes. Then, wells washed 3 times with PBS, tapped to remove residual liquid, and dried at room temperature. A mixture of 80% ethanol and 20% acetone was then added, and measurement of attachment determined by absorbance at 595 nm.

### Quellung reaction and microscopy

Polysaccharide capsule was visualized by microscopic examination of pneumococci after treatment with type-specific antibody (Statens Serum Institute, Copenhagen) as described previously [25]. Briefly, overnight cultures, grown in serum-medium with or without NE, were smeared onto a slide and air-dried. This was then covered with a coverslip containing 10 μl of 1% (w/v) methylene blue and 10 μl type specific anti-capsular antibody (Statens Serum Institute, Copenhagen, Denmark). The slide was examined by X1000 oil immersion microscopy.

### Statistics

Growth analyses were performed in triplicate and all experiments were performed on at least 3 separate occasions; unless stated otherwise, numerical data shown are expressed as mean +/- SD. Where appropriate, statistical analysis was first performed using one-way ANOVA and, if significant, an unpaired t-test. Statistical significance was indicated by a *P* value of less than 0 · 05.

## Results

### Norepinephrine stimulates *S. pneumoniae* growth and biofilm formation

**Figure 1:**
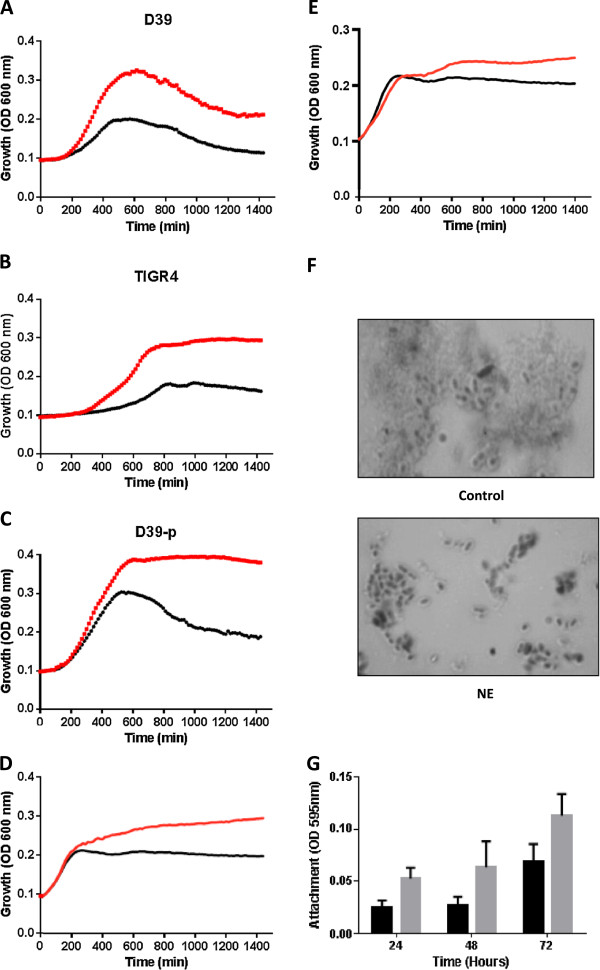
**NE stimulates pneumococcal growth and biofilm formation.** Panels **A-E** show the time course of growth of wildtype *S. pneumoniae* strain D39, TIGR4, and passaged D39 (D39-p) in serum-medium with (red line) and without (black line) the addition of 10 μM NE, epinephrine or dopamine. Panel **F** is a microscope image showing how NE increased clumping (cell-cell association) of D39 relative to the control, and also increased viability (shown by reduction in cellular lysis debris). Panel **G** shows the initial attachment of wildtype D39 after 24, 48 and 72 hrs incubation in serum-medium +/-10 μM NE. Attachment was measured as described in Materials and Methods; key: black bar (control); grey bar (NE); P < 0.05 for the collective values of all the NE vs. Control data points. Similar results were also seen with TIGR4 (data not shown). For all experiments, n = 3.

Wildtype *S. pneumoniae* strain D39, routinely propagated *in vitro* or its mouse passaged stock, and the TIGR4 strain were inoculated into serum-medium (50% Sicard [26] and 50% serum-SAPI, a host-like serum-supplemented minimal medium); serum was included to simulate *in vivo* conditions [2,8,23]. A time course of growth in serum-medium in the presence and absence of the catecholamine is shown in Figure 1, which reveals that the cultures grew significantly better (P < 0.01) when NE was present, indicating that all of the pneumococcal strains were stress hormone responsive. The passaged D39 showed overall greater growth levels (Figure 1C) relative to non-passaged bacteria with or without NE. Also, the decline in stationary phase optical density observed in the inotrope supplemented culture of the non-passaged D39 was not present in the NE-stimulated passaged strain. In these experiments we used 10 μM NE, a concentration which is attainable *in vivo*[13]. However, dose responses analyses showed that lower levels of 5 μM were equally stimulatory as 10 μM, which is a concentration attainable *in vivo*[13]. Interestingly, higher levels such as 50 μM produced no greater increases in cell numbers than the 10 μM NE addition (data not shown). We also found that the pneumococcus responds to other catecholamine stress hormones, and that addition of 10 μM dopamine or epinephrine were also stimulatory to growth (Figure 1D and E).

The ability of infectious bacteria to attach to surfaces, self-associate and form a biofilm is an aspect of virulence that is particularly important in the development of respiratory infections. It was found in the examination of the NE-treated cultures that the peumococcus displayed a different morphology to the un-supplemented control. Figure 1F is a light microscopy image of a quellung reaction assay [25] which shows that for D39 relative to the control, NE had no obvious effect on capsule formation, but did appear to reduce levels of cellular debris suggesting an enhancing effect on cell viability. What is also apparent in Figure 1F is the presence of NE increased clumping (cell-cell association) of the bacteria, which is an important intermediate stage in bacterial biofilm formation. We therefore investigated the effects of NE exposure on D39 attachment, the initial step in formation of a biofilm. Figure 1G shows over the course of a 3 day incubation in serum-medium that NE consistently enhanced the attachment of the wildtype D39 (p < 0.05). Very similar results were also found for strain TIGR4 (data not shown).

### The mechanism of pneumococcal catecholamine growth induction

**Figure 2:**
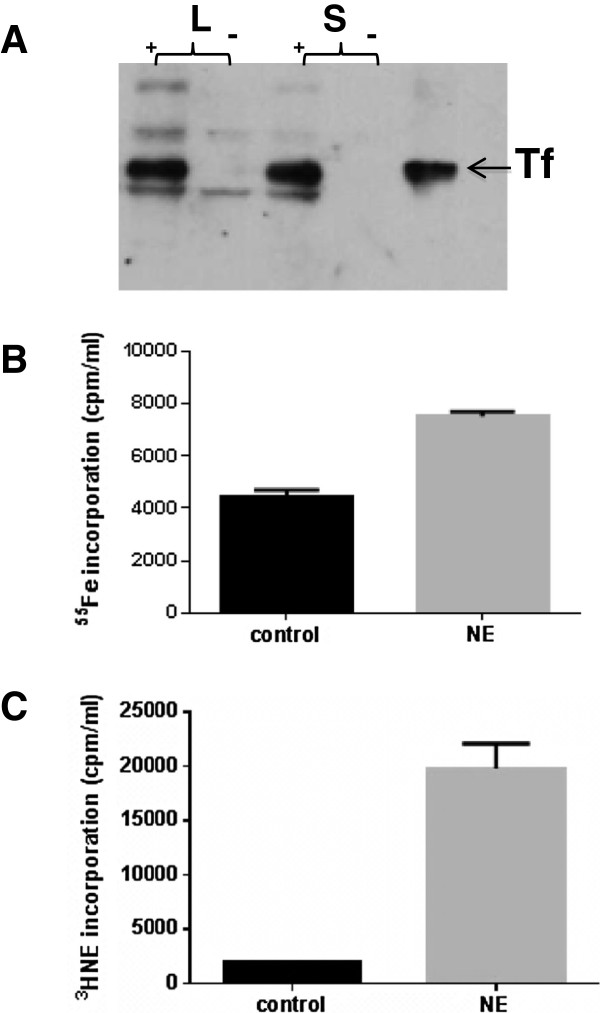
**Mechanism of norepinephrine growth induction.** Panel **A** is a western blot of Tf binding to D39 cultures (+) compared with non-Tf supplemented control cultures (-); the influence of growth phase on Tf binding is also shown: log-phase (L), stationary phase (S). Tf represents purified transferrin and is the positive control, indicated by the arrow. Panel **B** shows that uptake of Tf-complexed Fe (in the form of ^55^Fe) is increased in the presence of NE, P < 0.05; Panel **C** shows ^3^H-NE is internalised by D39. The values shown in panels **B** and **C** are means of triplicate counts; n = 3.

We have shown that catecholamines can stimulate bacterial growth by enabling access to the iron within host Fe binding proteins, such as transferrin [2,5,23,27]. Inotropes achieve this by virtue of having a catechol moiety, which can both bind and reduce ferric iron. This reduction to Fe(II) weakens the iron binding affinity of the transferrin, allowing bacteria to then uptake the released iron by either ferric or ferrous uptake systems [5]. Previous work has shown that *S. pneumoniae* can utilize ferric and ferrous iron salts, and host Fe sources such as haemoglobin, and haemin, but to a lesser extent the iron within Tf [28-30]. In terms of the growth stimulation seen in Figure 1, Figure 2A reveals that Tf is bound by *S. pneumoniae* in what appears to be a growth-phase independent manner. Figure 2B shows that incubation of the bacteria in serum-medium with Tf containing radiolabelled Fe (^55^Fe-Tf) allowed the pneumococcus to obtain normally sequestered host Fe in higher amounts if the stress hormone was present. We also investigated if the NE was taken up by the pneumococcus during catecholamine growth induction by including ^3^H-NE in the serum-medium. Figure 2C shows that ^3^H-NE was internalised, and that addition of non-labelled NE increased radiolabelled inotrope uptake (P < 0.05), possibly due to the capacity of the NE to increase cell numbers. Mechanistically, this indicates that inotrope-mediated Fe delivery from Tf and internalisation of the catecholamine are the probable explanations for the growth induction in serum-medium demonstrated by NE in Figure 1.

### *pspA* and *pspC* are involved in pneumococcal catecholamine inotrope responsiveness

**Figure 3:**
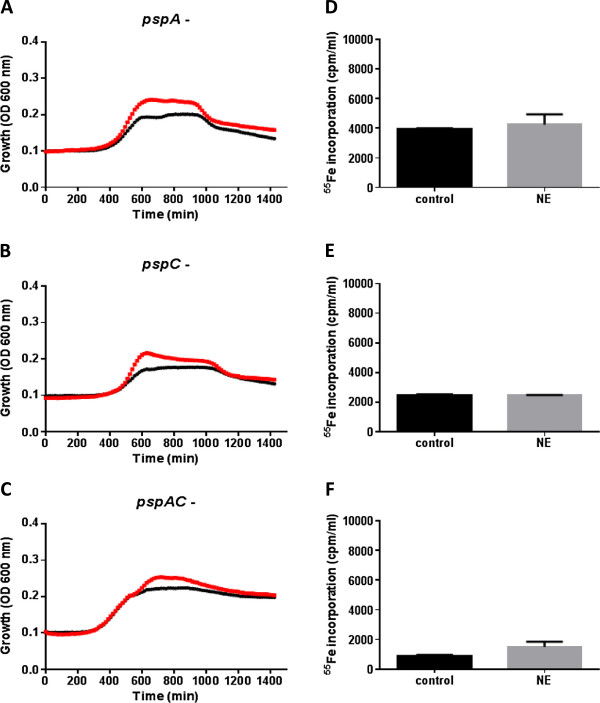
**Role of the PspA and PspC in pneumococcal catecholamine responsiveness.** Panels **A**-**C** show the time course of growth of *pspA*, *pspC* and *pspAC* mutants in serum-medium in the absence (black line) or presence of 10 μM NE (red line). Panels **D**-**F** shows the *pspA* (*pspA*^*-*^), *pspC* (*pspC*^*-*^) and *pspAC* (*pspAC*^*-*^) mutant uptake of Tf-complexed Fe (in the form of ^55^Fe) +/- NE; note that the wildtype D39 uptake of Tf-Fe in the presence of NE is shown in Figure 2B. Values shown in panels **D**-**F** are means of triplicate counts; for all experiments, n = 3.

The question that arose from the data in Figures 1 and 2 was what bacterial elements were involved in the mechanism by which NE facilitated growth increases in the pneumococcus, and so the role of key pneumococcal surface proteins, PspA and PspC, in the catecholamine growth induction mechanism was investigated. These mutants were chosen because previous studies had shown the involvement of PspA and PspC in pneumococcal persistence within the host, such as attachment, prevention of complement deposition and factor H recognition [19,20,31]. In addition PspA was shown to be important for binding the host Fe protein lactoferrin [32]. Hence, we constructed *pspA, pspC,* and *pspA* and *pspC* double mutants in the D39 pneumococcal strain and analysed their response to NE. Figures 3A-C show that in marked contrast to wildtype D39 (which is shown in Figure 2B), the *pspA* and *pspC* mutants, singly or in combination, showed no significant growth induction by the catecholamine.

To understand what the mechanism of this lack of response might be, we compared the ability of the *psp* mutants with wildtype D39 to acquire Fe from ^55^Fe-labelled transferrin in the presence and absence of NE. Figure 3D-F shows that in the absence of the catecholamine the *psp* mutants were able to uptake some ^55^Fe-iron, but unlike the parent strain (Figure 2B), were unable to utilise the NE to obtain higher levels of the Tf-complexed ^55^Fe, with the effect most strikingly seen in the double *psp* mutant which was overall severely compromised in its ability to obtain Fe from Tf (Figure 3F). We investigated if this was due to impaired binding of Tf, as PspA is a lactoferrin binding protein [32]. We conducted similar experiments to those used in Figure B for the *pspA, pspC,* and *pspA* and *pspC* mutants, but to our surprise binding of Tf was no less than that of the wildtype (data not shown).

**Figure 4:**
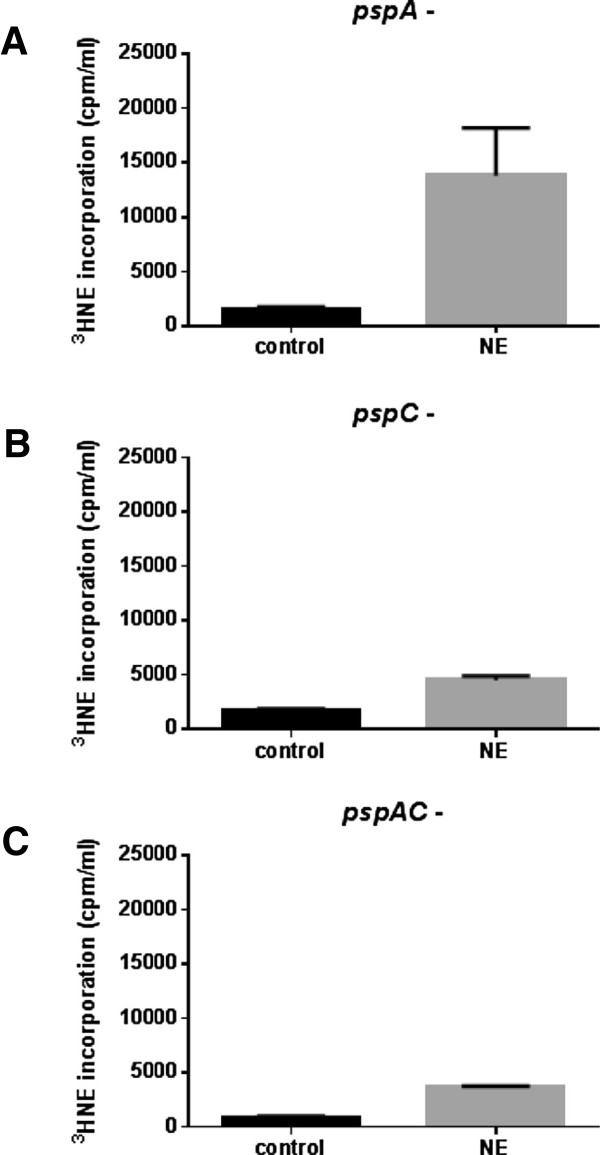
**Involvement of PspA and PspC in norepinephrine uptake.** Panels **A**-**C** show ^3^H-NE uptake *pspA*, *pspC* and *pspAC* mutants after 24 hrs incubation in serum-medium as described in Methods in the presence (grey) and absence of NE (black); P < 0.05. Values shown are means of triplicate counts; for all experiments, n = 3.

We showed in Figure 2C that NE was internalised by wildtype pneumococcus during the catecholamine growth induction, and since the *pspA* and *pspC* appeared to be non-NE responsive in the growth context, we also investigated if these proteins were in some way involved in the inotrope uptake. Figure 4 shows the internalisation of radiolabelled ^3^H-NE by the single and double *pspA* and *pspC* mutants, grown in serum-medium in the presence and absence of added NE. For the *pspA* mutant, the presence of unlabelled NE stimulated uptake of the ^3^H-NE to about 25% less than that shown by wildtype D39 (Figures 2C and 4A). In contrast, inactivation of *pspC* resulted in ^3^H-NE internalisation levels of >75% less than that of wildtype. The *pspA* and *pspC* double mutant showed similar uptake levels to the single *pspC* mutant. The data in Figures 3 and 4 collectively shows that the PspA and PspC appear to be integral elements in the mechanism by which NE induces *S. pneumoniae* growth in serum based medium.

### Norepinephrine modulates pneumococcal metabolism and virulence gene expression

We also examined in wildtype D39, and the *pspA* and *pspC* single mutants the effects of NE exposure on the expression of 14 functionally diverse genes involved in pneumococcal metabolism and virulence in order to identify genes involved in stress hormone responsiveness of *S. pneumoniae*. In wildtype D39 the expression of 9 genes manifested greater than two-fold changes (Table 2), the commonly agreed level of significance, although sometimes differences of less than two-fold are known to be biologically important. The genes with increased expression included *comX*, a response regulator for genetic competence, which is also involved in biofilm formation [33], consistent with our finding that NE increases pneumococcal attachment. Also showing up regulation was the SPD_0939: Rgg family transcriptional regulator, the members of which are involved in oxidative stress response, biofilm formation, quorum sensing and virulence [34,35]. In addition, differential expression of genes involved in polysaccharide degradation and sugar utilization (N-acetyl hexosaminidase, β-galactosidase galactokinase and pyruvate oxidase) as well as iron transport, *piuA,* were observed [36]. This is significant as *in vivo*, the concentration of free sugars are known to be low in the respiratory tract, forcing the pneumococcus to rely on host glycoproteins such as mucin, and the carbohydrates bound by host proteins such as Tf to satisfy essential carbon needs [37]. Others and we have demonstrated previously that glycosidases, such as neuraminidases and galactosidases are highly important for pneumococcal colonization and invasion [38].

**Table 2:** ***S. pneumoniae *** **gene expression in the presence of norepinephrine**

**Transcriptional regulators**	**Function**	**Fold change in expression**		
		**D39**	***pspA***^**-**^	***pspC***^**-**^
***comX *** **(SPD_0014)**	Transcriptional regulator ComX1	5.33 (0.15)	0.85 (0.03)	0.92 (0.09)
***rgg *** **(SPD_0144)**	Transcriptional regulator	1.72 (0.12)	0.40 (0.06)	1.13 (0.15)
***ritR *** **(SPD_0344)**	DNA-binding response regulator	0.77 (0.04)	0.73 (0.02)	0.97 (0.04)
***rgg*/*mutR *** **(SPD_0939)**	Transcriptional regulator	28.4 (2.2)	0.79 (0.06)	1.09 (0.13)
**Sugar hydrolases**				
***strH *** **(SPD_0063)**	N-acetyl hexosaminidase	6.56 (0.30)	0.6 (0.02)	0.97 (0.04)
***bga3 *** **(SPD_0065)**	β-galactosidase	21.4 (2.0)	0.78 (0.04)	1.1 (0.05)
***nanB *** **(SPD_1499)**	Neuraminidase B	22.39 (1.10)	1.23 (0.12)	0.78 (0.08)
***nanA *** **(SPD_1504)**	Neuraminidase A	10.77 (0.89)	1.44 (0.07)	0.98 (0.04)
**Cation metabolism**				
***piuA *** **(SPD_1652)**	Iron-compound ABC transporter	6.08 (0.20)	0.27 (0.03)	1.02 (0.03)
***psaA *** **(SPD_1463)**	ABC transporter substrate-binding protein	1.22 (0.04)	0.48 (0.03)	1.04 (0.03)
**Sugar metabolism**				
***pflB *** **(SPD_0420)**	Pyruvate formate lyase	0.59 (0.03)	0.89 (0.02)	1.03 (0.06)
***spxB *** **(SPD_0722)**	Pyruvate oxidase	0.16 (0.07)	0.69 (0.03)	0.84 (0.12)
***galK *** **(SPD_1634)**	Galactokinase	25.36 (2.39)	0.78 (0.04)	0.92 (0.07)
**Oxidative stress response**				
***tpxD *** **(SPD_1464)**	Thiolperoxidase	0.66 (0.07)	0.74 (0.06)	0.81 (0.1)

The relative gene expression levels of wildtype D39 and the *pspA* and *pspC* mutants was calculated from 3 independent experiments and standard deviation is indicated in parenthesis. The expression of target genes was normalised to the housekeeping gene *gyrB*.

We also show in Table 2 the catecholamine gene expression profiles of the *pspA* and *pspC* mutants. What is striking is that mutating *pspA* and *pspC* appears to have blocked NE-induced elevations in expression of many of the genes which in wild type D39 showed a NE-induced increase. For instance, expression of the *nanA* and *nanB* neuraminidases, which were up-regulated 22 and 10-fold-fold by NE in wildtype D39, were un-induced in the *pspA* and *pspC* mutants. Expression of the NE-responsive transcriptional response regulator *rgg/mutR*, which showed a 28-fold enhancement by NE in wildtype, was baseline in both mutants, as was also the response regulator *comX*. These results mirror strikingly the growth and NE uptake profiles shown in Figure 2.

## Discussion

Nasopharyngeal colonization is the first step of invasive pneumococcal disease [1]. However, it is not known what triggers the transition from colonization to invasiveness. Our on-going work on pneumococcal biology indicates that environmental factors, such as changing oxygen concentration, differences in metal and sugar composition of tissues, can have a fundamental impact on pneumococcal virulence [39,40]. However, although these environmental factors are important, they do not explain fully what triggers the sudden change from colonization to invasiveness. Therefore, we investigated whether other host factors, such as stress hormones, might be important for transition of the pneumococcus from commensal to pathogen [2]. The reason for this hypothesis stems from the rapid change in the concentration of stress hormones due to physical and emotional stress, from stress hormones’ adverse effect on immune system function [3], and from the microbial ability to recognize and process human stress hormone signals [2].

In this study we showed that *S. pneumoniae* responds to levels of catecholamine found within the circulation of inotrope-medicated patients [13] with increased growth and virulence, which could have a major impact on the progression of pneumococcal infection or transmission to new hosts. Many predisposing factors for pneumococcal diseases including emotional and cold stress, and overcrowding are known to increase stress hormone levels. In addition, catecholamine inotropes are administered up to 50% of patients in intensive care unit (ICU) [41], and up to 56% of patients with pneumococcal pneumonia are admitted to ICU [41]. Hence, in addition to endogenously produced stress hormones, pneumococci are exposed to externally applied catecholamine inotropes. Growth stimulation of *S. pneumoniae* came about due to the inotrope providing essential Fe for growth from the host iron binding protein transferrin, which was directly bound by the bacteria. Interestingly, the supposedly simple in function PspA and PspC surface proteins were found to play a major role in NE mediated growth induction. When the genes for PspA and PspC were mutated, the ability of *S. pneumoniae* to utilize the additional Fe provided from transferrin by the catecholamine was reduced. The uptake of the radiolabelled NE was similarly reduced. Also, mutating *pspA* and *pspC* appeared to block NE-effects on gene expression, which agrees well with the non-growth responsiveness observed. Why PspA and PspC should be so important in mediating catecholamine responsiveness in the pneumococcus is unclear. The two proteins are important in virulence as they have been shown to play a pivotal role in the inhibition of complement-mediated opsonization [42,43], in prevention of lactoferrin killing [44], and in facilitating the microbe’s attachment to the respiratory tissues and the brain microvascular endothelium [19,30-32]. PspA is also known to bind to lactoferrin [32]. In addition, PspA and PspC have been shown to elicit protective antibody response against invasive pneumococcal infection, hence they are considered to be promising vaccine candidates [31]. Although their contribution to *S. pneumoniae*-host interaction is well studied, comparatively little is known about their role in pneumococcal physiology. Previously, using recombinant PspA and a strain mutated in *pspA*, it was shown that PspA, but not PspC, is responsible for pneumococcal binding to human lactoferrin, which was suggested to be important to overcome the iron limitation at mucosal surfaces [44,45]. Contrary to previous reports [32,45], in this study we consistently demonstrated that *S. pneumoniae* could bind to transferrin, and acquire iron from this glycoprotein, and that uptake of Fe from Tf was enhanced when NE was present. The reason for this discrepancy could be due to different culture conditions, and detection technology used for transferrin binding. For example, unlike Hakansson *et al*., (2001) [45] we used a serum based medium to prepare pneumococcal cultures, which can affect the synthesis of proteins involved in binding to Tf. Currently, the mechanism of PspA and PspC mediated pneumococcal response to NE is not known and so defining how PspA and PspC are mediating catecholamine responsiveness is a current focus of our laboratories. However, based on the available data it is clear that these surface proteins are required for recognition and/or internalisation of NE since the mutation of *pspA* or *pspC* abolished NE responsiveness, reduced NE uptake and blocked catecholamine-induced gene responsiveness. This clearly indicates that the proteins encoded by these genes may be acting as a sensor molecule. It is not surprising that both PspA and PspC are involved in stress hormone mediated effects in *S. pneumoniae* given these proteins are coded by paralogous genes, and previous studies have demonstrated their involvement in similar biological events [31,42,43]. In future experiments, we plan to investigate to which downstream targets PspA and PspC relay NE mediated messages.

A recent study by Marks *et al.*[12] showed that NE treatment of biofilms formed in *vitro*, and *in vivo* in the nasopharynx leads to dispersion of *S. pneumoniae*, and the dispersed cells display distinct phenotypic traits that are different from those of both biofilm and broth-grown planktonic bacteria. The dispersed pneumococci were shown to have differential virulence gene expression, and had a significantly increased ability to disseminate and cause infection in the middle ear, lungs, and bloodstream. Our results are consistent with Marks *et al*., [12] in that the pneumococcus responds to NE, and that treatment with the catecholamine leads to differential gene expression. On the other hand, contrary to the Marks *et al*. study, who used biotic surfaces to determine NE’s role in pneumococcal dispersion from biofilms, our results show that in host like serum-containing media the catecholamine aggregates the pneumococci and promotes biofilm formation on abiotic surfaces. The reason for this seeming discrepancy could be due to methodological differences and also be attributed to NE’s possible dual function in biofilm formation. In other words, NE can initially promote bacterial biofilm formation (our current study) and after a certain stage in the infection process, depending on the microbial growth phase, may also promote dispersion of the pneumococci [12]. Interestingly, a recent paper from Gonzales *et al.*[46] found that addition of a non-therapeutic level of NE (100 μM) stimulated several-fold increases in growth but in contrast to our data, had an inhibitory effect on pneumococcal biofilm formation, as measured by attachment to host cells. Therefore, it is clear that further work is required to understand this differential effect of the catecholamine on biofilm formation.

In this study NE mediated Fe uptake from Tf was identified as the mechanism responsible for the observed growth effect of NE in serum based media. However, our gene expression analysis in wild type D39 shows that NE has an even wider effect on pneumococcal physiology. For example, the expression of genes coding for glycosidases (*nanA*, *nanB*, *bgaC* and *strH*), which are responsible for deglycosylation of host glycans and play important role in pneumococcal colonization and invasiveness [36,37], were significantly upregulated in the presence of NE. Moreover, differential expression of genes involved in transcriptional regulation (SPD_0939), competence development (*comX*), galactose metabolism (*galK*), and iron transport (*piuA*) was also detected, indicating the comprehensive effect of NE on pneumococcal metabolism. Currently it is not known how the pneumococcus detects and processes stress hormone signals, though there is a clear involvement of PspA and PspC in the response mechanism. Therefore, investigating the underlying genetic mechanisms for detection and processing of catecholamine signals is a priority. Also, in this study we found that the pneumococcus responds similarly to a variety of catecholamine stress hormones (NE, as well as dopamine and epinephrine), which is in contrast to the situation demonstrated in *Mycoplasma hypopneumoniae*[47]. This finding is also of clinical significance as 300 μM epinephrine may be administered directly to ventilated patients to reduce airway inflammation [8].

Bacteria have evolved mechanisms to sense the changes in the stress hormone levels using receptors, which appear to be specific and able to differentiate between different stress hormones [2,4,48]. Using α and β receptor antagonists, we showed the presence of putative adrenergic and dopaminergic receptors in three Gram-negative bacteria: *Escherichia coli*, *Salmonella enterica* and *Yersinia enterocolitica*[49]. Our results demonstrated that catecholamine- induced growth in these bacteria could be blocked by catecholamine α-receptor antagonists, but not by antagonists for β adrenergic receptors. But, so far, no comprehensive study has been conducted to investigate proteins responsible for stress hormone recognition in Gram positive bacteria. Identification of such receptors in the pneumococcus would enhance our understanding of *S. pneumoniae*-host interactions and may offer alternative therapeutic options against pneumococcal diseases.

## Conclusions

A clinically attainable level of NE stimulated pneumococcal growth via a mechanism involving inotrope-delivery of transferrin-iron and internalisation of the inotrope. NE also markedly increased *S. pneumoniae*-biofilm formation. Gene profiling showed that norepinephrine significantly enhanced expression of genes involved in central metabolism and host colonisation. Analysis of the response of the pneumoccal *pspA* and *pspC* mutants to the stress hormone showed them to have a central involvement in the catecholamine response mechanism. Collectively, our results suggest that inotrope-pneumococcal interactions may be a contributory factor for the development of *S. pneumoniae*-associated pneumonia.

## Competing interests

The authors declare that they have no competing interests.

## Authors’ contributions

HY and PF co-designed the study, and carried out some of the experiments. SMS co-designed the study and with contributions from FA carried out most of the experiments. All authors contributed to the writing of the final manuscript. All authors read and approved the final manuscript.

## Authors’ information

Primrose Freestone and Hasan Yesilkaya are senior authors.
